# Ewing Sarcoma of the Lung: Imaging of a Rare Tumor

**DOI:** 10.7759/cureus.32395

**Published:** 2022-12-11

**Authors:** Ilianna Tsetsou, Hippocrates Moschouris, Nektarios Spanomanolis, Evridiki Soumpourou

**Affiliations:** 1 Department of Radiology, Tzaneio General Hospital of Piraeus, Piraeus, GRC; 2 Department of Interventional Radiology, Tzaneio General Hospital of Piraeus, Piraeus, GRC; 3 2nd Department of Internal Medicine, Tzaneio General Hospital of Piraeus, Piraeus, GRC

**Keywords:** transthoracic biopsy, extraosseous ewing sarcoma, primary pulmonary ewing sarcoma, ewing sarcoma (es), extraskeletal ewing's sarcoma

## Abstract

Primary pulmonary Ewing sarcoma is an extremely rare tumor of neuroectodermal tissue. In this article, we report on the case of a 45-year-old female who presented in the emergency department with shortness of breath and night fever. Radiologic findings suggested a massive pulmonary mass and a metastatic liver lesion. The diagnosis of Ewing sarcoma was established through a percutaneous biopsy of the lung mass and liver lesion. We highlight the importance of considering a broad differential diagnosis for a large pulmonary mass in order to lead to a prompt diagnosis and treatment.

## Introduction

Primary pulmonary Ewing sarcoma (PPES) is an exceptionally rare tumor with a poor prognosis [1]. Similar to other pulmonary tumors, imaging evaluation of PPES is based on computed tomography (CT), magnetic resonance (MR), and 18-fluorodeoxyglucose-positron emission tomography (FDG-PET). In this report, we present a case of PPES in an adult female patient, and we focus on the relevant imaging findings.

## Case presentation

A 45-year-old Caucasian female presented to our emergency department complaining of shortness of breath and low-grade night fever for two months. She had no history of smoking and reported a medical history of hypothyroidism and hysterectomy for uterine fibroids.

Vital signs were normal and physical examination revealed dullness on percussion and diminished respiratory sounds over the right hemithorax. Laboratory workup showed mild normocytic anemia (hematocrit: 32.7%), increased erythrocyte sedimentation rate (87 mm/h), mildly increased aspartate transaminase (45 U/I), gamma-glutamyl transferase (56 U/l), and lactate dehydrogenase (LDH) (1194 U/l). Serum tumor markers (carcinoembryonic antigen (CEA), alpha-fetoprotein (a-FP), cancer antigen (CA 125), CA 15.3, CA 19.9) were within normal range.

**Figure 1:**
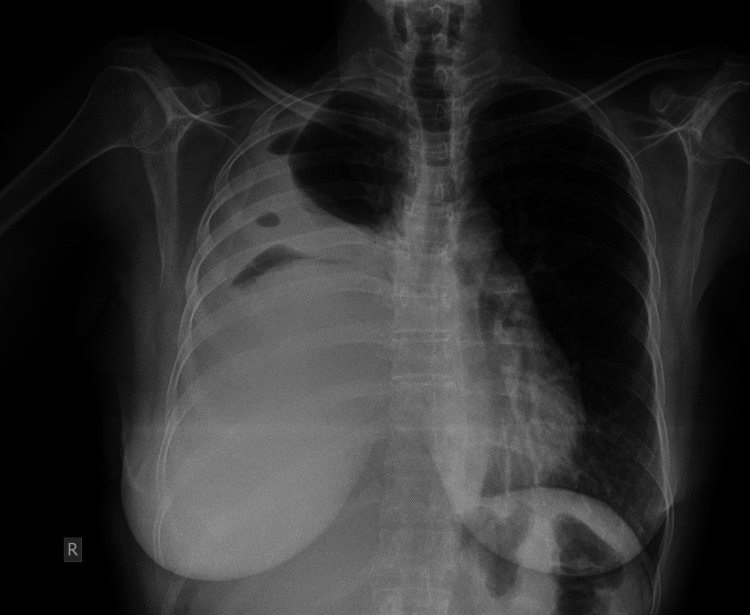
Chest X-ray Anteroposterior chest X-ray showing opacification of 3/4th of the right hemithorax, with a laterally rising border and loculated air pockets within the opacification, suggestive of pleural effusion and loculated pneumothorax

An anteroposterior erect chest X-ray showed an opacity occupying most of the right hemithorax that prompted further evaluation to initially rule out pleural effusion (Figure 1).

**Figure 2:**
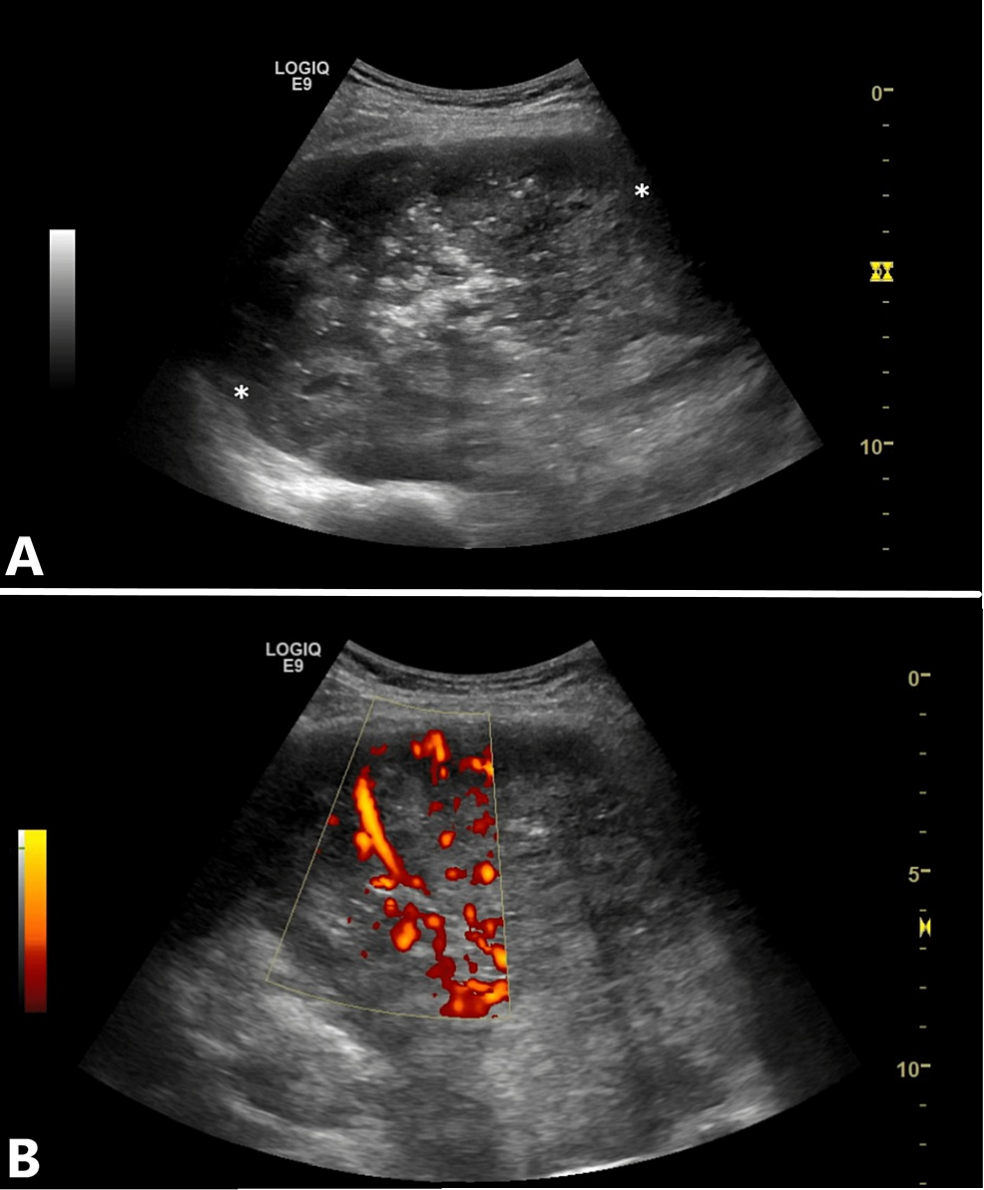
Transthoracic ultrasound A: Grayscale US showing a predominantly hypoechoic mass with amorphous calcifications (asterisks); B: Color Doppler (B-Flow) depicting rich vascularity

Transthoracic ultrasound (US) was performed using a General Electric Logiq E9 unit (GE Healthcare, Milwaukee, WI) and a multifrequency (2-5 MHz) curved array transducer. The US examination revealed a predominantly hypoechoic mass, measuring approximately 14x12 cm, with central branching and irregular echogenic foci (Figure 2A). Color Doppler and B-Flow imaging detected a relatively rich vascularity with multiple, tortuous arteries surrounding avascular areas. There were no signs of direct thoracic wall invasion. An abdominal US scan was subsequently performed, which revealed a 7.5 x 5 cm iso- to slightly hyperechoic lesion with a hypoechoic rim in segment 8 of the liver.

**Figure 3:**
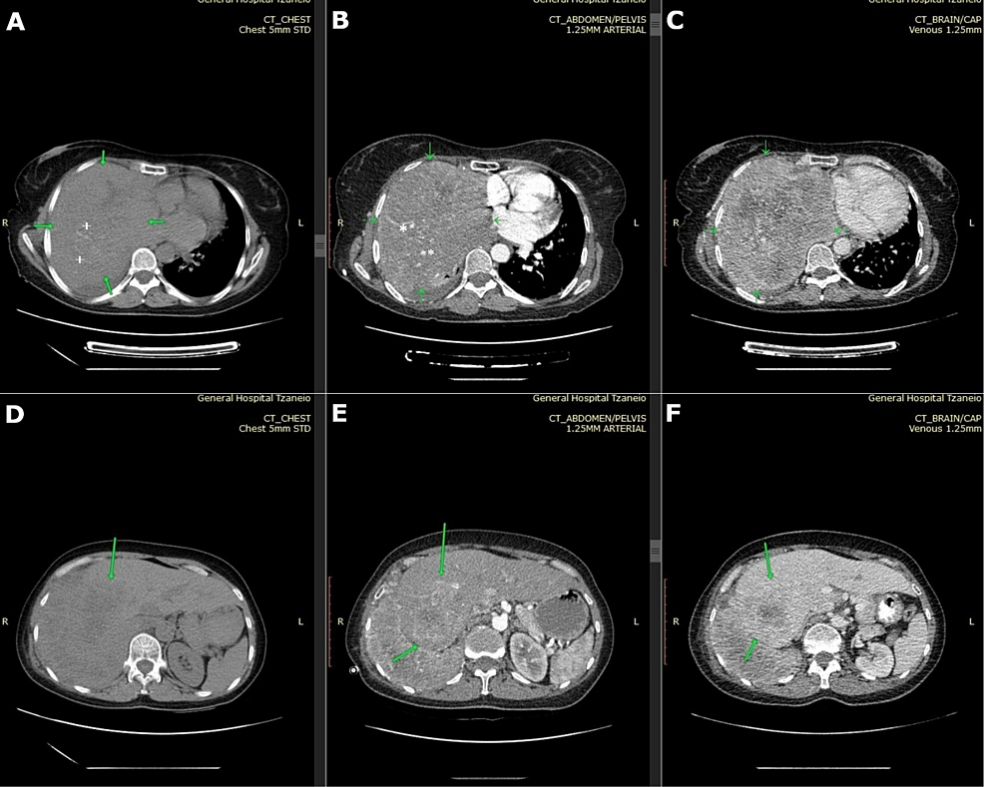
CT findings 3A-3C: Axial chest CT (A is non-contrast, B is arterial phase, and C is portal venous phase) at the level of the heart. A large heterogenous mass with central amorphous calcifications (A, crosses) and neovascularity (B, asterisks) is occupying the right hemithorax. The arrows show the mass margins. 3D-3F: Axial abdominal CT (same phases as above). The heterogeneous mass is shown with arrows at segment 8 of the liver. It has similar imaging findings to the lung mass.

An abdominopelvic and thoracic CT scan with intravenous contrast was also performed for further evaluation of the masses (Figure 3). CT better delineated the extent of the disease, confirming a large right pulmonary mass, measuring approximately 21 x 16 cm, with central vascularity and amorphous calcifications, accounting for some of the echogenic foci seen on ultrasound. It also ruled out thoracic wall invasion. Additional findings included a small pleural effusion, compressive atelectasis of the upper right lung, and dilated collateral vessels in the axillary region. The known liver lesion in segment 8 showed similar imaging characteristics. Both masses were indicative of malignant lesions, and the liver mass was considered a metastasis from a primary lung malignancy. No other organ involvement was noted.

**Figure 4:**
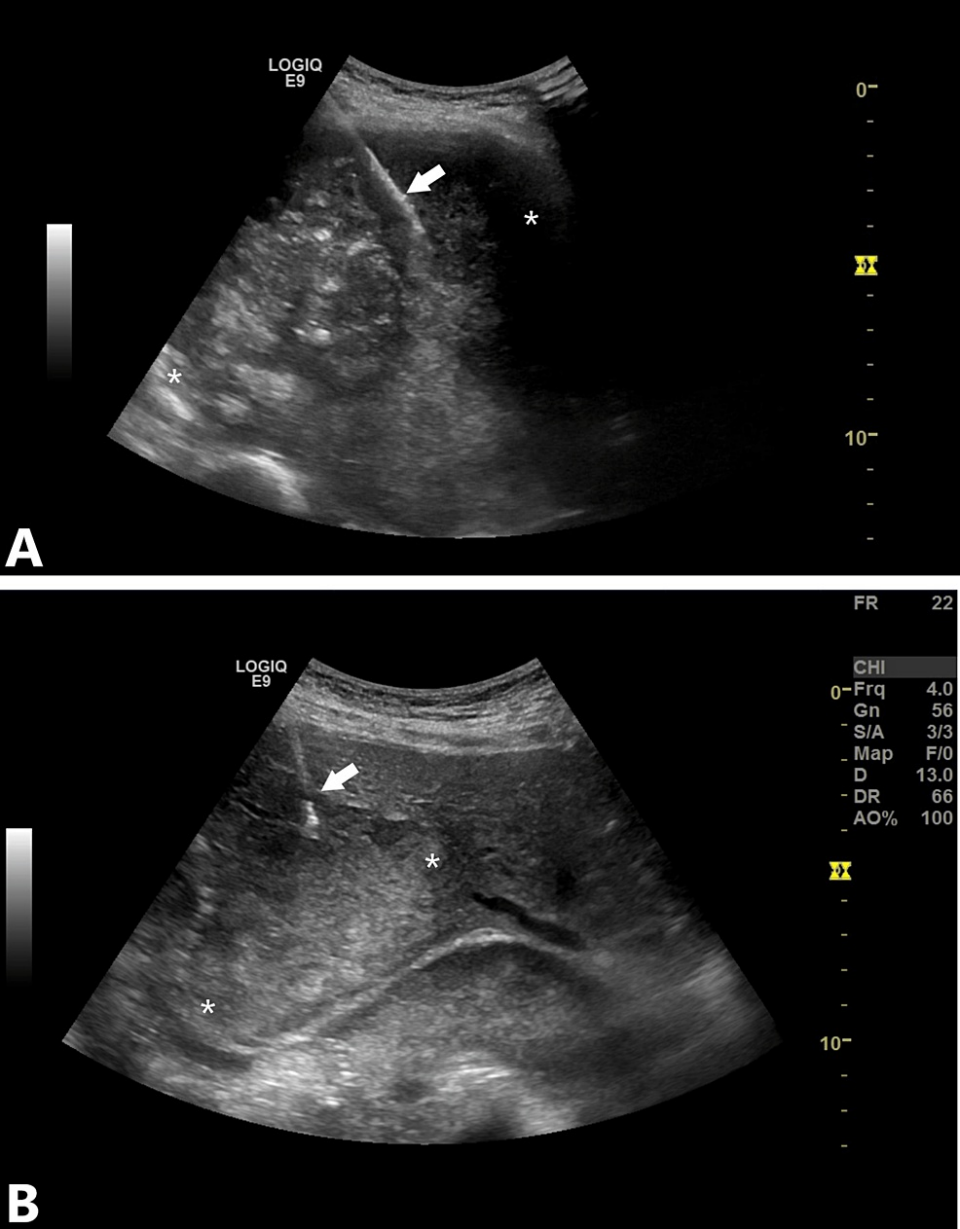
Percutaneous biopsy Percutaneous biopsy of the lung (A) and liver (B) mass. In both pictures, the arrows show the biopsy needle and the asterisks show the margins of the masses.

A percutaneous Tru-Cut biopsy of both lesions was performed under ultrasonographic guidance, with a free-hand technique and a semiautomatic, 18-gauge biopsy gun (DSGBL 18/10, Tsunami Medical, Modena, Italy) (Figure 4). The procedure was well-tolerated and uneventful.

**Figure 5:**
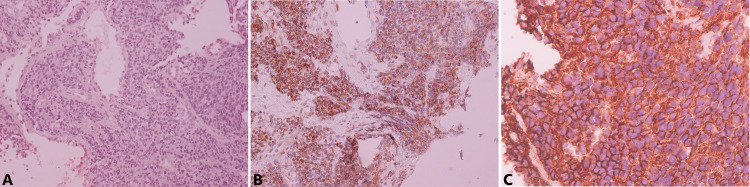
Histology A: Histology that shows small round cells with indistinct pale eosinophilic cytoplasm (hematoxylin-eosin stain, magnified x200); B, C: Immunohistochemically, the cells are positive for BCL-2 (B) and CD99+ (C) (magnified X200 and x400, respectively)

The histology of both specimens showed diffuse proliferation of small round cells with indistinct pale eosinophilic cytoplasm (Figure 5). Immunohistochemically, the tumor cells were diffusely positive for CD99+ and ERG+ and positive for BCL2+, synaptophysin, and vimentin. Staining for FLI-1, desmin, WT-1, CK8/18, NF, and STAT-6 was negative. Genetic analysis showed translocation in the EWSR1 gene. The aforementioned findings supported the diagnosis of PPES with liver metastasis.

The patient was subsequently urgently referred to a specialist center and treated with a combination of multiagent cytotoxic chemotherapy (cyclophosphamide, vincristine, and doxorubicin, alternating with ifosfamide and etoposide) but unfortunately succumbed to progressive disease two and a half months after the initial diagnosis.

## Discussion

First described by James Ewing et al. [2], the Ewing sarcoma family of tumors encompasses a group of malignancies that include Ewing sarcoma of the bone, extraosseous Ewing sarcoma, peripheral primitive neuroectodermal tumor (PNET), and thoracopulmonary PNET, also known as Askin tumor [3]. PPES is rare, with only a handful of cases reported so far (Table 1) [4-19].

**Table 1:** Reports of patients with PPES over the past 10 years PPES: primary pulmonary Ewing sarcoma

Reference	Year	Sex	Age	Location	Outcome (follow-up)
Ichiki	2012	Male	42	Right lower lobe	Alive (6 months)
Andrei	2013	Male	31	Lingula	Died (3 years)
Hwang	2014	Female	41	Left lower lobe	Alive (4 years)
Deokar	2015	Female	30	Right lung	Died (15 days)
Hirano	2015	Female	19	Left lower lobe	Alive (4 years)
Mizuguchi	2016	Male	70	Left upper lobe	Died (4 months)
Shet	2016	Male	23	Right upper lobe	Alive (6 months)
Ekin	2019	Male	28	Lingula	-
Zhang	2019	Male	33	Right and left lung	-
Anne	2020	Male	36	Left lower lobe	Alive (18 months)
Dharmalingam	2020	Male	46	Right upper lobe	-
Wu	2020	Male	33	Right upper lobe	-
Gupta	2020	Male	42	Right lung	Died (8 days)
Sohn	2020	Female	49	Right lower lobe	Alive (2 years)
Ata	2021	Male	16	Left lung	Died (15 days)
Ling	2021	Female	15	Right lung	-
Tungate	2022	Female	74	Left upper lobe	Alive (2 years)

It was first reported by Hammar et al. in 1989 [20]. The definitive diagnosis is made histologically. It belongs to the group of small round-cell sarcomas. CD99+ is used as a diagnostic marker by immunohistochemistry in approximately 95% of Ewing sarcomas. Molecular detection of EWSR1 rearrangements confirms the diagnosis [21].

Imaging plays an important role in the evaluation of this rare clinical entity. Extraosseous Ewing sarcomas, such as PPES, tend to manifest as a non-calcified unilateral thoracic mass with well-defined margins. They usually appear paravertebrally and there is no involvement of the bone marrow [22]. Extraskeletal sarcomas usually displace the surrounding tissues instead of infiltrating them [23]. Radiological workup includes transthoracic US, CT, bone scintigraphy and/or FDG-PET, and, in certain cases MR, to evaluate the extent of disease and possible metastases [21].

Transthoracic US can be initially utilized to evaluate a mass adjacent to the chest wall. It can aid significantly in the diagnosis of thoracic tumors by visualizing possible invasion of adjacent tissues and describing their morphologic characteristics. Additionally, percutaneous lung biopsies guided by US are followed by fewer complications when compared to other guiding methods, such as CT, and are more convenient in terms of both time and resources [24]. US also aids in the early identification of iatrogenic complications such as pneumothorax [25].

In CT, PPES most commonly appears as a large mass that displaces adjacent structures. It is usually inhomogeneous due to rapid growth, with cystic degeneration and mixed internal attenuation [23]. Ipsilateral pleural effusion or calcifications may also be present [26]. A typical finding in FDG-PET is increased uptake of FDG. Therefore, FDG-PET can be used to detect bone marrow metastases [21].

## Conclusions

Diagnosis of PPES is particularly challenging. Usually, imaging alone cannot be diagnostic due to the rarity of this disease and the non-specific findings. However, it is of vital importance to assess the extent of the disease and rule out other pathologies. Histopathology and molecular testing are necessary to establish the final diagnosis. Nevertheless, the radiologist should keep in mind and consider a broad spectrum of differential diagnoses when coming across a large, primary lung mass with well-defined margins and mass effect on surrounding tissues.
